# Ureteroarterial fistula: Late infectious common iliac artery pseudoaneurysm formation following successful endovascular stenting and literature review

**DOI:** 10.1002/iju5.70002

**Published:** 2025-04-29

**Authors:** Shinnosuke Hiruta, Toshiaki Shinojima, Masao Takahashi, Takao Nonaka, Harunobu Matsumoto, Hirotaka Asakura

**Affiliations:** ^1^ Department of Urology Saitama Medical University Moroyama Saitama Japan; ^2^ Department of Radiology Saitama Medical University Moroyama Saitama Japan; ^3^ Department of Vascular Surgery Saitama Medical University Moroyama Saitama Japan

**Keywords:** infectious pseudoaneurysm, ureteroarterial fistula, vascular stent graft

## Abstract

**Introduction:**

The long‐term prognosis of endovascular stenting for ureteroarterial fistulas is not always favorable. We present a case in which endovascular repair of a ureteroarterial fistula led to the development of an infectious iliac artery pseudoaneurysm that required open vascular graft replacement 1 year later.

**Case presentation:**

A 38‐year‐old woman with radiation‐induced vesicovaginal fistula and bilateral ureteral stenosis underwent urinary diversion using an ileal conduit. During left ureteral stent exchange, a ureteroarterial fistula occurred on the left side and was successfully treated with vascular stent grafting. One year later, gross hematuria recurred, requiring open surgical intervention because of the formation of an infectious pseudoaneurysm near the aortic bifurcation.

**Conclusion:**

Several patients treated with stent grafting for ureteroarterial fistulas require subsequent reintervention. Urologists managing patients with ureteroarterial fistulas should collaborate closely with interventional radiologists and vascular surgeons to ensure comprehensive care.

**Keynote message**

Several patients treated with stent grafting for UAF require subsequent reintervention. Close collaboration between urologists and vascular surgeons is essential to ensure comprehensive care.

**Abbreviations & Acronyms**
A: alive
CIA: common iliac artery
CT: computed tomography
D: dead
F‐F: femorofemoral
IC: ilial conduit
ISVR: in‐situ vascular reconstruction
Nx: nephrectomy
PCN: percutaneous nephrostomy
SG: stent graft
UAF: ureteroarterial fistula
UCN: ureterocutaneostomy
UKN: unknown
US: ureteral stenting

## Introduction

Patients with UAF may present with potentially life‐threatening hematuria that requires prompt diagnosis and treatment.^1^ Coordinated management of the urinary and vascular systems is critical; however, the optimal approach to urinary tract management in UAF has yet to be definitively established. Herein, we present a case of endovascular repair of a UAF without coordinated urinary drainage resulting in the formation of an infectious iliac artery pseudoaneurysm.

## Case presentation

A 38‐year‐old woman with a history of radiation therapy for cervical cancer was referred for the treatment of a large radiation‐induced vesicovaginal fistula and bilateral lower ureteral stenosis. Urinary diversion with an ileal conduit was performed; however, left ureteral stenosis persisted and necessitated drainage using a 7Fr ureteral single J stent extending beyond the stoma, which was exchanged every 6–8 weeks.

Two years later, the patient presented with hematuria and left flank pain, leading to hemorrhagic shock. Under UAF diagnosis, a GORE® VIABAHN® stent graft was successfully deployed in the left CIA. The left ureteral single J stent was removed based on the expectation that the fistula would spontaneously close due to ureteral occlusion. However, the patient experienced recurrent episodes of left pyelonephritis caused by the left ureteral stenosis. The patient refused nephrostomy catheter placement despite repeated counseling.

**Fig. 1:**
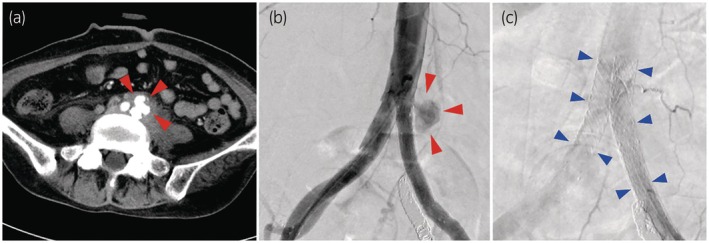
(a) CT angiography shows contrast spillage at the left proximal CIA (red arrows) at the proximal end of the stent graft placed for the left UAF 1 year before. (b) Angiography shows a left CIA aneurysm (red arrows). (c) Implantation of stents (blue arrows) in the bilateral CIA using the so‐called kissing stent technique, with a slight overlap within the distal aorta to create a “kissing” configuration at the aortic bifurcation. This technique allows endovascular treatment of lesions involving near the aortic bifurcation.

One year later, the patient presented with gross hematuria and right flank pain. CT revealed right hydronephrosis and a blood clot in the right lower ureter. Elevated serum creatinine levels indicate postrenal dysfunction, likely due to the development of right UAF. A right percutaneous nephrostomy was performed, followed by stent graft placement in the right iliac artery. However, the presence of right UAF could not be definitively confirmed during the procedure. Although renal function and right flank pain improved postintervention, hematuria from the stoma and the pain in the lower back persisted. CT performed 3 weeks later revealed a newly formed pseudoaneurysm in the proximal part of the left CIA (Fig. 1a). This was considered to be within the area of prior whole‐pelvic 30 Gy irradiation, which included the common iliac lymph nodes. Thus, we hypothesized that radiation‐induced arterial damage, exacerbated by recurrent left upper urinary tract infection, caused the iliac pseudoaneurysm. Additionally, it was hypothesized that the damaged artery had not only formed a fistula connecting to an unidentified location in the urinary tract, resulting hematuria, but also contributed to persistent lower back pain from the impending rupture. Considering the complications of previous radiation and surgeries, interventional radiology was selected as the preferred treatment over open surgery. The right nephrostomy tube was removed because the CT scan showed delayed arterial bleeding at the nephrostomy tract. After selective embolization of the right renal artery, stent grafts were implanted into the bilateral CIA (Fig. 1b,c). This procedure effectively resolved the pseudoaneurysm, hematuria, and back pain. The patient underwent left nephrostomy for local infection control and was subsequently discharged.

**Fig. 2:**
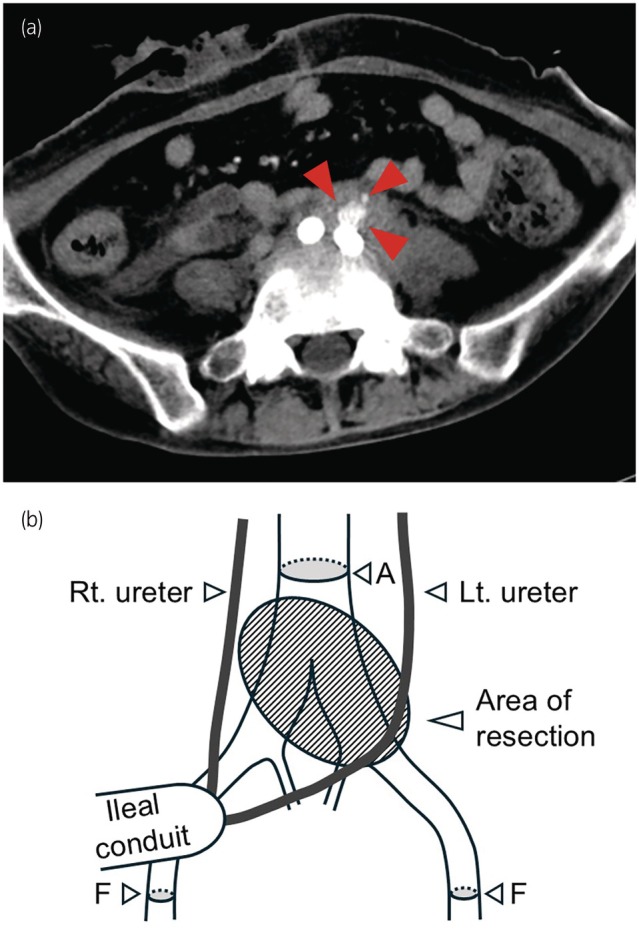
(a) CT angiography shows a recurrent pseudoaneurysm in the left CIA (arrows). (b) Schematic of the procedure. Resection of the infected arterial wall was initiated after clamping the aorta and bilateral femoral arteries. The resected area includes the aortic bifurcation and surrounding fibrous tissue. A Y‐graft is anastomosed proximally to the aorta (A) and distally to the bilateral femoral arteries (F).

Fourteen days later, the patient presented with a new onset of lower back pain, nausea, and hematuria. CT revealed the recurrence of the pseudoaneurysm at the original site (Fig. 2a). Subsequently, vascular surgeons urgently performed resection of the infected arterial wall, extending from the distal aorta to the bilateral common iliac arteries (Fig. 2b). Although the left ureter was resected along with the adherent infected arterial tissue, the presence of a right UAF was not evident, even during laparotomy. A potential explanation for the right UAF misdiagnosis could be the retrograde entry of bleeding from a left UAF, overlooked despite its recurrence, into the right lower ureter via the proximal end of the ileal conduit. Following the resection of the infected tissues, vascular grafting extended from the aorta distal to the inferior mesenteric artery to the bilateral femoral arteries. The stent grafts removed from the left iliac artery exhibited no signs of infection, such as pus. However, *Pseudomonas aeruginosa*, which had been repeatedly identified in the patient's urine, was present in a culture of the resected arterial tissue. These findings indicated that the arterial wall infection originated from the urinary tract and not from the stent graft. The patient was followed for 10 months with no recurrence of hematuria and underwent regular left nephrostomy exchanges.

## Discussion

UAF is a rare but potentially life‐threatening condition by massive bleeding. The pathophysiology involves the development of abnormal communication between the ureter and an artery, most commonly the iliac artery.^1^ Ischemic and fibrotic damage of the ureter caused by surgery and radiation leads to the fixation of the ureteral wall to an iliac artery. Ureteral stents can exacerbate the condition by causing friction and pressure on the ureteral wall, ultimately leading to UAF.^2^ Although open surgery and endovascular treatments are viable options for UAF management, endovascular therapy is increasingly favored because of its less invasive nature.^3^ Despite this shift, open surgery remains necessary in cases of uncontrolled local infection, previous failure of endovascular stenting, or an infected stent graft.^4^

As summarized in Table 1, our case demonstrates that the long‐term prognosis of endovascular stenting is not always favorable. One single‐center study reported the incidence of reintervention for hematuria and other complications was 36% (4/11) within 1 year,^5^ while another reported that the conversion rate to open surgery was 70% (14/20) within the same period.^4^ After stent graft placement, our patient did not undergo aggressive drainage using a nephrostomy catheter for a urinary tract infection on the same side as the UAF, which may have led to an uncontrolled local infection and necessitated open surgery. Therefore, a literature review was conducted to determine the prognostic impact of upper urinary tract treatment following endovascular treatment for UAF. Forty‐three cases from 36 studies, detailing both urinary treatment and follow‐up, were sourced from PubMed between 2013 and 2023 and are summarized in Table 2. Among the six patients who did not undergo coordinated urinary tract interventions during the stent graft implantation, only three patients retained a functioning kidney on the side of the UAF during the implantation. Notably, two of these three patients subsequently underwent either prophylactic nephrectomy or ureteral stenting as part of their follow‐up care. Therefore, monitoring patients without providing therapeutic drainage to the urinary tract is not considered standard practice. Instead, ureteral stenting or nephrostomy is typically considered standard options for such patients. However, 8 of 22 patients (36%) with nephrostomy management eventually required additional vascular interventions during a median follow‐up period of 10 months (range 1–66 months). This incidence is not significantly different from that observed in patients managed with ureteral stents (38%, 5/13). Considering the high recurrence rate of vascular lesions, particularly when the kidney on the UAF side remains functional, close collaboration between urologists and vascular surgeons is essential to ensure comprehensive care.

**Table 1:** Summary of clinical course

	Time from first presentation (m, month; w, week)				
	0	24 m	37 m	37 m + 3w	37 m + 5w
Disease	Vesicovaginal fistula with bilateral ureteral stenosis	Lt. UAF	Rt. Hydronephrosis with gross hematuria	Infectious pseudoaneurysm of proximal lt. CIA	Recurrence of pseudoaneurysm
Vascular treatment urinary	None	Lt. iliac artery SG	Rt. iliac artery SG	Bil. CIA kissing stent	Explantation of SG; Y‐graft bypass
Urologic treatment	Urinary diversion; Lt. single J ureteral stenting	None	Rt. nephrostomy	Lt. nephrostomy	Lt. nephrostomy

**Table 2:** Upper urinary tract management following endovascular treatment for ureteroarterial fistula (UAF). The details of the references are given in the Supporting Information

Age/sex	Predisposing factors			Upper urinary tract management following SG placement	Subsequent treatment or outcome/months after SG placement	Follow‐up (months)	Status
	Urinary diversion	Radiation	US				
54/M	−	−	−	None		12	A
68/F	−	−	−	None^†^		6	A
43/F	−	+	+	None^‡^		8	A
64/M	−	+	+	None^‡^		7	D
63/M	−	−	+	None	Prophylactic Nx/6	6	A
55/M	−	−	−	None	US/1	2	A
37/F	−	+	+	PCN	F‐F bypass/12	18	A
55/F	IC	−	+	PCN	Explantation of SG; F‐F bypass/3	15	A
79/F	−	+	+	PCN	Endovasuclar restenting/8	10	D
45/F	−	+	+	PCN	Explantation of SG; F‐F bypass/<1	1	A
36/F	−	+	+	PCN	Explantation of SG; F‐F bypass/6	30	A
UKN	IC	UKN	+	PCN	Endovasuclar restenting/UKN	4	D
UKN	UCN	UKN	+	PCN	Endovasuclar restenting/UKN	7	D
84/F	−	−	+	PCN	Explantation of SG/3.5	3.5	D
45/F	−	−	+	PCN		2.5	A
70/F	−	+	+	PCN		2	A
73/F	−	+	+	PCN		4	D
73/F	IC	+	+	PCN		25	A
57/F	−	+	+	PCN		63	A
46/F	−	−	+	PCN		2	D
69/F	−	+	+	PCN		1	A
UKN	IC	UKN	+	PCN		10	A
UKN	UCN	UKN	+	PCN		8	A
UKN	UCN	UKN	+	PCN	Nx/UKN	32	A
66/F	IC	+	+	PCN	Contralateral UAF/24	66	A
82/M	IC	+	+	PCN	Removal of PCN tube/2	12	A
84/F	−	−	−	PCN	Removal of PCN tube/3	24	A
56/F	−	−	−	PCN	Removal of PCN tube/<1	1.5	A
78/M	UCN	−	+	US	Endovasuclar restenting/6	8	D
52/F	−	+	+	US	Explantation of SG; ISVR/12	96	A
51/F	−	+	+	US	Ureteral ligation; PCN; explantation of SG/24	48	A
71/F	IC	+	+	US	Endovascular restenting; PCN/<1	1	A
75/F	IC	+	+	US	Nx; explantation of SG; ISVR/<1	1	A
84/F	−	−	+	US		12	A
55/F	−	+	+	US		6	A
80/F	−	+	+	US		30	A
68/M	IC	+	+	US		12	A
UKN	−	UKN	−	US		12	A
58/F	IC	+	+	US	Prophylactic Nx; urinary diversion/4	4	A
50/F	−	+	+	US	Contralateral UAF/3	15	A
48/F	IC	+	+	US	Contralateral UAF/6	6	A
61/M	−	+	−	Nx		22	A
63/F	−	−	+	Nx		6	A
38/F^§^	IC	+	+	None	PCN; explantation of SG; Y‐graft bypass/14	24	A

^†^ Non‐functioning kidney on the side of UAF.

^‡^ A Kidney on the side of UAF had been removed unnecessarily due to hematuria.

^§^ Our case.

## Conclusion

Several patients treated with stent grafting for UAF require subsequent reintervention, regardless of coordinated urinary tract management. Urologists managing patients with UAF should collaborate with vascular surgeons to ensure comprehensive care.

## Author contributions

Shinnosuke Hiruta: Writing – original draft. Toshiaki Shinojima: Conceptualization; writing – review and editing. Masao Takahashi: Writing – review and editing. Takao Nonaka: Writing – review and editing. Harunobu Matsumoto: Writing – review and editing; supervision. Hirotaka Asakura: Supervision.

## Conflict of interest

The authors declare no conflict of interest.

## Approval of the research protocol by an Institutional Reviewer Board

Not applicable.

## Informed consent

Written informed consent was obtained.

## Registry and the Registration No. of the study/trial

Not applicable.

## Supporting information


Table S1.
